# The effectiveness of a low-intensity problem-solving intervention for common adolescent mental health problems in New Delhi, India: protocol for a school-based, individually randomized controlled trial with an embedded stepped-wedge, cluster randomized controlled recruitment trial

**DOI:** 10.1186/s13063-019-3573-3

**Published:** 2019-09-18

**Authors:** Rachana Parikh, Daniel Michelson, Kanika Malik, Sachin Shinde, Helen A. Weiss, Adriaan Hoogendoorn, Jeroen Ruwaard, Madhuri Krishna, Rhea Sharma, Bhargav Bhat, Rooplata Sahu, Sonal Mathur, Paulomi Sudhir, Michael King, Pim Cuijpers, Bruce F. Chorpita, Christopher G. Fairburn, Vikram Patel

**Affiliations:** 1 Sangath, C-1/52, 1st Floor, Safdarjung Development Area, New Delhi, Delhi 110016 India; 2 Department of Clinical, Neuro and Developmental Psychology, Faculty of Behavioural and Movement Sciences, Vrije Universiteit Amsterdam, van der Boechorstraat 1, 1081 BT Amsterdam, The Netherlands; 3 School of Psychology, University of Sussex, Falmer, Brighton, BN1 9RH UK; 4 MRC Tropical Epidemiology Group, London School of Hygiene & Tropical Medicine, Keppel Street, London, WC1E 7HT UK; 5 Amsterdam UMC, Amsterdam Public Health research institute, Vrije Universitiet, Van der Boechorststraat 7, 1081 BT Amsterdam, The Netherlands; 6 GGZ inGeest Specialized Mental Health Care, Oldenaller 1, 1081 HL Amsterdam, The Netherlands; 7 Department of Clinical Psychology, National Institute of Mental Health and Neurosciences, Hosur Road, Bangalore, Karnataka 560029 India; 8 Division of Psychiatry, Faculty of Brain Sciences, University College London, Maple House, 149 Tottenham Court Rd, London, W1T 7NF UK; 9 Department of Clinical, Neuro and Developmental Psychology, Amsterdam Public Health research institute, Vrije Universiteit Amsterdam, van der Boechorstraat 1, 1081, BT Amsterdam, The Netherlands; 10 Department of Psychology, University of California, Los Angeles, 3227 Franz Hall, Los Angeles, CA 90095-1563 USA; 11 University Department of Psychiatry, Warneford Hospital, Oxford, Oxford, OX3 7JX UK; 12 Department of Global Health and Social Medicine, Harvard Medical School, 641, Huntington Avenue, Boston, MA 02115 USA

**Keywords:** Mental health, Problem-solving, Psychological intervention, Stepped-wedge trial, Adolescents, Schools, Randomized controlled trial, Low- and middle-income countries, India

## Abstract

**Background:**

Conduct, anxiety, and depressive disorders account for over 75% of the adolescent mental health burden globally. The current protocol will test a low-intensity problem-solving intervention for school-going adolescents with common mental health problems in India. The protocol also tests the effects of a classroom-based sensitization intervention on the demand for counselling services in an embedded recruitment trial.

**Methods/design:**

We will conduct a two-arm, individually randomized controlled trial in six Government-run secondary schools in New Delhi. The targeted sample is 240 adolescents in grades 9–12 with persistent, elevated mental health symptoms and associated distress/impairment. Participants will receive either a brief problem-solving intervention delivered over 3 weeks by lay counsellors (intervention) or enhanced usual care comprised of problem-solving booklets (control). Self-reported adolescent mental health symptoms and idiographic problems will be assessed at 6 weeks (co-primary outcomes) and again at 12 weeks post-randomization. In addition, adolescent-reported distress/impairment, perceived stress, mental wellbeing, and clinical remission, as well as parent-reported adolescent mental health symptoms and impact scores, will be assessed at 6 and 12 weeks post-randomization. We will also complete a parallel process evaluation, including estimations of the costs of delivering the interventions.

An embedded recruitment trial will apply a stepped-wedge, cluster (class)-randomized controlled design in 70 classes across the six schools. This will evaluate the added effect of a classroom-based sensitization intervention over and above school-level sensitization activities on the primary outcome of referral rate into the host trial. Other outcomes will be the proportion of referrals eligible to participate in the host trial, proportion of self-generated referrals, and severity and pattern of symptoms among referred adolescents in each condition. Power calculations were undertaken separately for each trial. A detailed statistical analysis plan will be developed separately for each trial prior to unblinding.

**Discussion:**

Both trials were initiated on 20 August 2018. A single research protocol for both trials offers a resource-efficient methodology for testing the effectiveness of linked procedures to enhance uptake and outcomes of a school-based psychological intervention for common adolescent mental health problems.

**Trial registration:**

Both trials are registered prospectively with the National Institute of Health registry (www.clinicaltrials.gov), registration numbers NCT03633916 and NCT03630471, registered on 16th August, 2018 and 14th August, 2018 respectively).

**Electronic supplementary material:**

The online version of this article (10.1186/s13063-019-3573-3) contains supplementary material, which is available to authorized users.

## Background

Adolescence is a critical period for the prevention and treatment of mental health problems. Around 10% of adolescents experience a mental disorder [1] and about half of all mental disorders have their onset by the mid-teens, rising to three-quarters by the mid-20s [2]. Effective early intervention is therefore vital to mitigate the substantial personal, familial, and societal costs of mental disorders [3]. Low- and middle-income countries (LMICs) are home to 90% of the world’s 1.3 billion adolescents, but there is a severe shortage of mental health services targeting this age group in most LMICs [4]. This includes India, which is home to one-fifth of the global population of adolescents. Resource constraints are compounded by low demand for mental health care and the scarcity of context-specific evidence on the effectiveness of interventions [5]. Although a robust body of research testifies to the treatability of adolescent mental disorders, mainly through psychological interventions such as cognitive behavioral therapy (CBT), the bulk of such evidence originates from high-income countries [6]. Generalizability of the existing evidence base to LMICs is further restricted by the widespread use of specialist providers in intervention trials, with supervision often provided directly by program developers [7].

Transdiagnostic approaches have been advocated as a means of providing more scalable psychological interventions [8], with emerging evidence (mainly from adult populations) supporting their use in LMICs [9, 10]. Transdiagnostic interventions recognize the considerable overlap that exists in the constituent elements of disorder-specific protocols and the abundance of shared risk and protective factors for psychopathology in general [11]. The available data suggest that transdiagnostic interventions may be comparable in effectiveness to their disorder-specific counterparts, although head-to-head comparisons are scarce [12]. There are also indications that transdiagnostic protocols may confer advantages in terms of improved efficiencies afforded by the parsimonious use of a single intervention framework for multiple problems [13], as well as meeting an expressed need among practitioners for therapies that are designed to fit ‘real-world’ settings where psychosocial complexity and comorbidity are commonplace [14].

The PRIDE (PRemIum for aDolEscents) research program involves linked studies in India with the goal to design and evaluate a scalable transdiagnostic intervention model that addresses common mental health problems (i.e., anxiety, depression, and conduct difficulties) in school-going adolescents. The public health importance of adolescent mental health has been recognized in the National Adolescent Health Program (the Rashtriya Kishor Swasthya Karyakram) [15]. PRIDE was initiated in response to these national and global priorities, and challenges, for improving the quality and coverage of adolescent mental health interventions. The process of aligning the global evidence base on youth psychotherapies with local evidence followed recommendations from an earlier research program (PREMIUM) on psychological intervention development in low-resource settings, which led to the design and demonstration of the clinical effectiveness of two brief psychological treatments for adult mental health problems [16–18].

Our formative and pilot studies have informed the design of a stepped-care architecture involving two interventions of incremental intensity [19–22]. The current trial protocol focuses on the first step: a low-intensity problem-solving intervention designed for delivery by non-specialist school counsellors. Problem solving is strongly represented in the global literature, where it is among the most commonly used practice elements in evidence-based mental health programs for children and adolescents [23, 24]. It has been applied successfully as the main element in other low-intensity psychological interventions in LMICs [25, 26]. The emphasis on problem solving also reflects the primacy of psychosocial factors in adolescents’ narratives around explanatory models of distress and help-seeking [21]. Our provisional theory of change for the intervention draws on evidence-based principles of stress and coping [27], such that the impact of an ecological stressor is assumed to be mediated by appraisals of the stressor and of the repertoire of available coping resources. Our problem-solving intervention can be considered transdiagnostic in the sense that a single procedure is assumed to have generalized benefits for a diversity of clinical presentations. Non-responders to this first-line intervention will be offered a more intensive and dynamic transdiagnostic treatment incorporating additional cognitive and behavioral procedures. The effectiveness of the second step will be evaluated in a separate randomized trial for which participants will be recruited from a different school cohort.

As well as shaping the design of the two intervention steps, formative and pilot work suggested a need for awareness generation around the topics of mental health and psychological help-seeking. We therefore developed a sensitization plan to address factors such as low mental health literacy and confidentiality concerns, which might otherwise impede the demand for school mental health services. In so doing, we noted the lack of consistent evidence for the effects of school-based and other youth-focused mental health sensitization interventions. Existing approaches have varied considerably in their design and intensity [28, 29] and their ability to increase demand from adolescents for mental health care has yet to be established [30, 31]. We therefore identified an opportunity to test an additional component of the PRIDE intervention architecture—a classroom sensitization session led by school counsellors—by embedding a recruitment trial within a host intervention trial [32]. Where applicable, the distinctive features of the two trials are presented sequentially, structured according to the Standard Protocol Items: Recommendations for Interventional Trials (SPIRIT) guidelines [33]. Shared features of the two trials (e.g., data management) are presented under unified headings.

## Objectives and hypotheses

### Embedded recruitment trial

The primary objective of this stepped-wedge, cluster randomized controlled trial is to evaluate the impact of a classroom sensitization session (intervention condition), over and above school-level sensitization activities (control condition), on the rate of referred adolescents (i.e., the proportion of adolescents referred as a function of the total sampling frame in each condition) into the host trial. The primary hypothesis is that the classroom-level sensitization intervention will be associated with a higher referral rate into the host trial compared with referrals arising from school-level sensitization activities in isolation. The secondary hypotheses are that, compared with the control condition, the intervention condition will be associated with a greater proportion of referred students who meet eligibility criteria for inclusion in the host trial (Table 1) and a greater proportion of students who self-refer. We will also explore whether there are any differences between conditions in terms of the severity of total symptoms and symptom subtypes presented by referred adolescents.

**Table 1:** Eligibility criteria for the host trial

Participant group	Inclusion criteria	Exclusion criteria
Adolescents	Eligible adolescent participants will be: i) Enrolled as a student in grades 9–12 (approximately 13–20 years in age) in one of the six collaborating schools; ii) Experiencing elevated mental health symptoms, based on response in the borderline or abnormal range using cut-offs derived from a normative school-based sample in India (≥ 19 for boys and ≥ 20 for girls) [34] on the adolescent-reported Total Difficulties score of the Strengths and Difficulties Questionnaire (SDQ) [35]; iii) Experiencing significant distress and/or functional impairment, based on response in the abnormal range (≥ 2) on the adolescent-reported SDQ Impact Supplement; iv) Experiencing difficulties for > 1 month, based on response to the adolescent-reported chronicity item of the SDQ Impact Supplement; and v) For adolescents under 18 years of age, able to provide informed assent to participate and supported by parental consent; or vi) For adolescents over 18 years of age, able to provide informed consent to participate	Adolescents will be excluded, if they: i) Require urgent medical or mental health care (defined as needing emergency treatment or in-patient admission); ii) Are currently receiving treatment for a mental health problem; iii) Received school counseling in the preceding 6 months as a participant in PRIDE pilot studies; iv) Exhibit difficulties in written and/or spoken Hindi that may impede their ability to participate fully in trial procedures; and/or v) Are unable to communicate clearly (e.g., due to a speech, learning or hearing disability)
Parents	Eligible parent participants will be: i) A primary parental caregiver or guardian for the index adolescent; ii) Able to provide informed consent for their own participation, and for the participation of the index adolescent (if aged under 18 years); and iii) If the index adolescent is aged age 18 years or older, permission for parental involvement has been provided by the adolescent	Parents will be excluded, if they: i) Are unable to communicate clearly (e.g., due to a speech or hearing disability); ii) Are unable to comprehend Hindi; and/or iii) Are intoxicated at the point of consent or assessment

### Host trial

The primary objective of this two-armed, parallel-design, individually randomized controlled trial is to evaluate the effectiveness of a low-intensity, problem-solving intervention (intervention arm) in reducing adolescent-reported mental health symptoms and idiographic problems at 6 weeks post-randomization, compared with enhanced usual care (control arm), for adolescents with common mental health problems. The primary hypothesis is that the problem-solving intervention will be superior to the control arm in reducing the severity of adolescent-reported mental health symptoms and idiographic problems at 6 weeks post-randomization.

The secondary objectives are:

- To evaluate the effectiveness of the intervention on adolescent-reported distress/functional impairment, perceived stress, mental wellbeing, and clinical remission
- To explore whether a theoretically informed a priori factor (perceived stress at 6 weeks) mediates the effects of the intervention on mental health symptoms and idiographic problems at 12 weeks
- To explore the effectiveness of the intervention on caregiver-reported adolescent mental health symptoms, associated distress/functional impairment, and adolescent-reported prosocial behavior
- To evaluate intervention delivery processes in order to assist in the interpretation of the trial results and to inform potential implementation of the PRIDE interventions on a wider scale
- To estimate the costs and cost-effectiveness of implementing the PRIDE interventions

## Methods/design

Methods are described according to SPIRIT guidelines [33]. Completed SPIRIT checklists for the two trials are provided as Additional files 1 and 2.

### Study setting

The two trials will be conducted in six Government-run secondary schools in New Delhi, India. The schools were purposively selected in consultation with the Department of Education, Government of New Delhi, to focus on relatively under-served, low-income communities. Of the six schools, three are boys’ schools, two are girls’ schools and one is co-educational. Each school contains grades 6–12, of which grades 9–12 will be the focus of this research. As of August 2018, there were 172 classes in grades 9–12 with a total student population of 8448 (ranging from 1050 to 1632 per school; mean = 1408; standard deviation (SD) = 225), including 4694 (56%) boys and 3754 (44%) girls.

### Participants

**Fig. 1:**
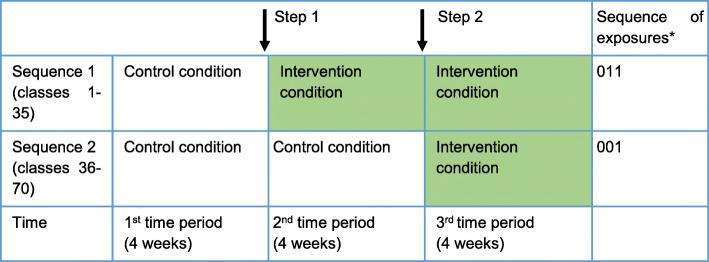
Illustration showing implementation of the control and intervention conditions in the embedded recruitment trial. The *white boxes* indicate the group of classes in the control condition and the *colored boxes* indicate the group of classes in the intervention condition. *0 = control condition; 1 = intervention condition

**Fig. 2:**
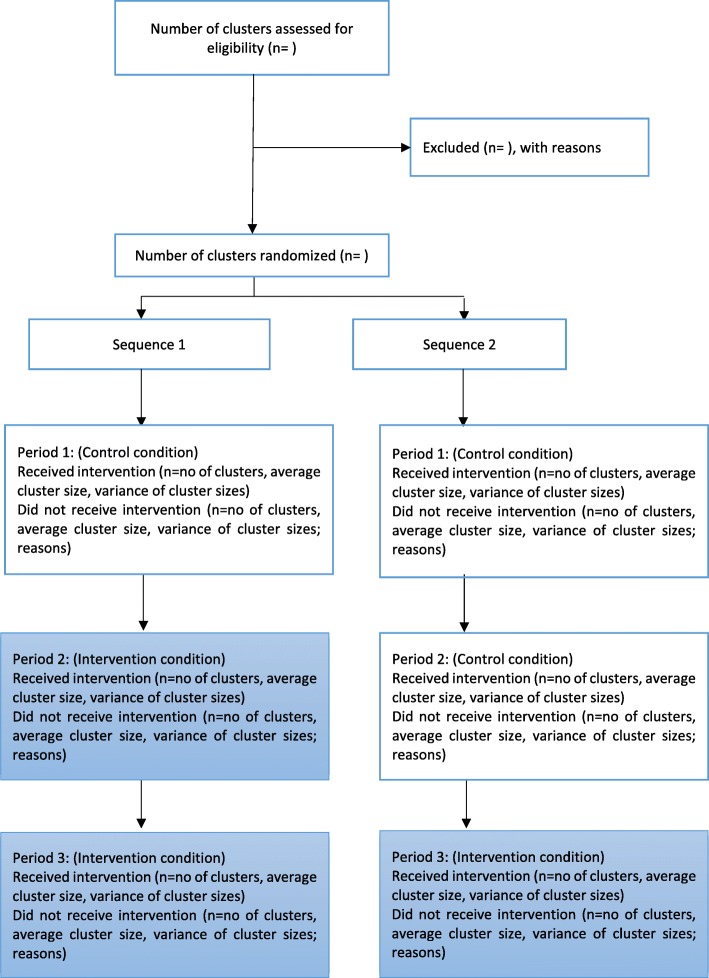
CONSORT flowchart for the embedded recruitment trial. The *white boxes* indicate the group of classes in the control condition and the *colored boxes* indicate the group of classes in the intervention condition

**Fig. 3:**
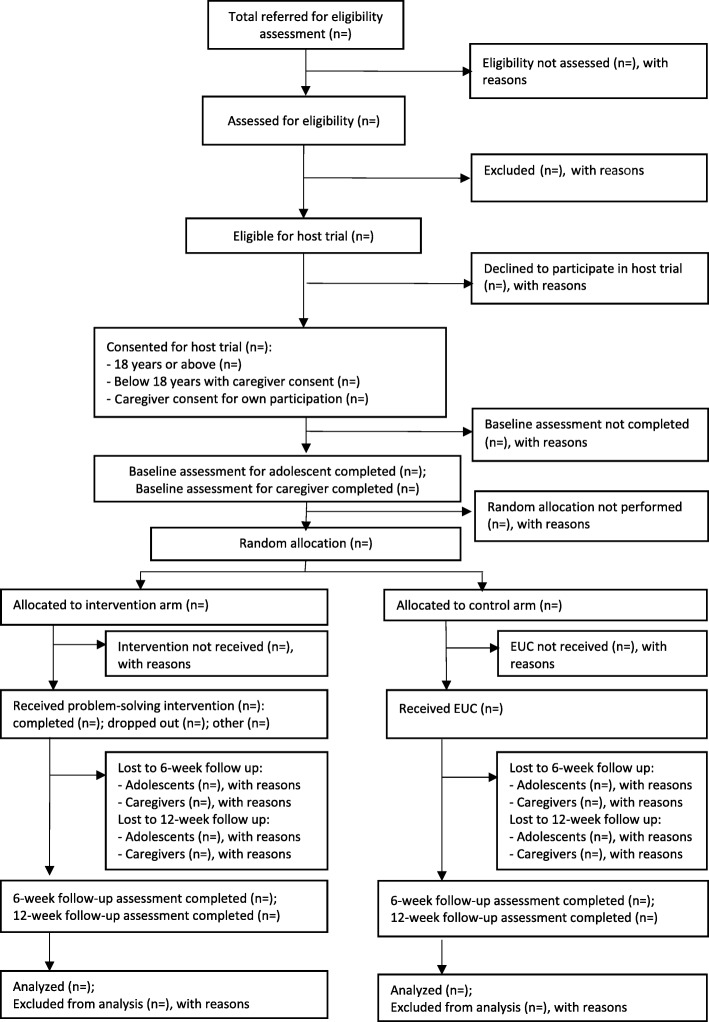
CONSORT flowchart for the host trial

Figures 1 and 2 summarize the participant timeline and flow for the embedded recruitment trial as per CONSORT guidelines for reporting stepped-wedge, cluster-randomized controlled trials [36]. Figure 3 presents the CONSORT diagram for the host trial [37].

#### Embedded recruitment trial

Seventy classes will participate in the embedded recruitment trial. These classes will be selected at random using computer-generated random numbers, stratified by school and grade, drawing from a pool of 118 eligible classes (excluding 54 classes that had received sensitization during earlier pilot work in these schools). The participating 70 classes will be randomized to receive the control and intervention conditions across two sequences. A small block size of 2 will be used to allocate the 70 classes across the two sequences in order to ensure balance, as the number of classes within each grade from the individual schools is relatively small. In the rare instance that a selected class has been dissolved or merged with another class, the next class in the random list will be included to replace the unavailable class. Each sequence will be implemented over three consecutive 4-week intervals (excluding holidays and exam breaks). Thus, each class will switch over from the control to the intervention condition at 4-week intervals, over two steps (Fig. 1). Schedules for sensitization in the allocated classes will be shared with the schools in advance to ensure access.

#### Host trial

Referrals to the host trial will be generated through a combination of self-referrals, teacher referrals, and referrals made by friends, siblings, and/or parents. These referrals will be drawn from the 70 classes sampled in the embedded recruitment trial, with additional participants recruited from the remaining 102 classes as needed. The precise schedule of recruitment activities in the latter classes will be calibrated according to referral patterns and caseload capacity for intervention providers in the various schools. When initiating a self-referral, students will have the option to either approach a counsellor directly or else post a completed referral form/written note in a secure drop-box. The school counsellor will also serve as a central point of contact for other potential referrers, and will offer referral forms on request. All referred adolescents will be followed up by a researcher and screened for eligibility to participate in the host trial (Table 1).

Consenting participants (see section on consent procedures below) will be enrolled by researchers and randomized to the intervention or the control arm after baseline outcome assessments are completed. Participants who are randomized to the intervention arm will be escorted by a researcher to meet the counsellor in an adjacent room/cubicle, ensuring efficient and discrete handover. The randomization list will be developed by an independent statistician (HW), applying stratification by school (and gender for the co-educational school) using randomly sized blocks of four or six. The randomization code will be concealed using sequentially numbered opaque sealed envelopes to maximize allocation concealment. Errors in randomization will be recorded and reported.

### Sample size and power calculations

#### Embedded recruitment trial

We based our power calculation on a within-period comparison [38] for a stepped-wedge design using Stata package “clustersampsi”. Based on pilot data, we anticipated referral rates of 5% and 15% on the control and intervention conditions, respectively, with an intra-cluster correlation coefficient (ICC) of 0.124. We assumed the same ICC for the between-time correlation given the short time period of follow-up. In practice, it may be smaller than 0.124 and both ICCs will be reported. Using these parameters, a sample size of 70 classes (average class size of 50 students) will have 92% power to detect a difference of 10 percentage points (treating the outcome as a binomial variable), at a significance level of 0.05.

#### Host trial

Sample size estimations were produced for two co-primary outcomes: severity of adolescent-reported mental health symptoms measured by the Total Difficulties score on the Strengths and Difficulties Questionnaire (SDQ) and severity of idiographic problems measured by the Youth Top Problems (YTP; Table 2). We based the estimations on two data sources. First, we obtained uncontrolled effect sizes (ES = Difference in means/pooled SD) for both co-primary outcomes from a group of 52 adolescents who received the problem-solving intervention during pilot work in the six secondary schools in New Delhi. Among these students, all of whom met the same baseline eligibility criteria as intended for the current trial, the mean SDQ Total Difficulties scores changed from 23.4 (SD 3.4) at baseline to 16.1 (SD 5.9) at the end of the intervention (ES = 1.4). The mean YTP scores for the same group changed from 5.6 (SD 2.0) at baseline to 2.9 (SD 2.6) at the end of the intervention (ES = 0.9). Second, we obtained a paired effect size on the SDQ Total Difficulties score from another cohort of 47 adolescents participating in a later phase of piloting, including 29 students who received the problem-solving intervention and 18 waitlisted controls (ES = 1.0). YTP data were unavailable for this second cohort. Effect sizes in trials are often smaller than in pilots so we conservatively hypothesized that our intervention would be associated with an ES = 0.5 on both the co-primary outcomes with 90% power. We assumed a 1:1 allocation ratio of individual participants within each of the six schools, loss to follow-up of 15% over 6 weeks (based on piloting), and a Bonferroni correction to adjust for multiple primary outcomes. Based on these assumptions, we determined that *N* = 240 participants would be required. This sample size also provides 80% power to detect an ES of 0.44.

### Interventions

#### Embedded recruitment trial

##### Intervention condition

This will comprise a one-off 30-min classroom session that is intended to improve understanding about signs and symptoms of mental health problems, raise awareness about the school counselling service, and generate demand for the service. The session will be delivered for individual classes (approximately 50 students per class) by a counsellor (drawn from the same group responsible for the problem-solving intervention in the host trial) with assistance from a researcher who will have additional responsibilities for processing referrals and conducting eligibility assessments. The classroom session will start with a short animated video (https://drive.google.com/file/d/1Y2NoMYf-NTjuNekYgxWZf7nNZIg88E98/view?usp=drivesdk) which provides age-appropriate information about types, causes, impacts and ways of coping with common mental health problems. The video will be followed by a guided group discussion, structured around a standardized script that builds on the topics covered in the video. In case of technical difficulties that may prevent the video from being shown, the counsellor will use a flipchart based on printed images from the video. At the end of the session, students will be handed a self-referral form which includes normalizing information and question-based prompts to assist with self-identification of mental health problems. Interested students can approach the facilitators immediately after the session with self-referral forms, or else deposit the forms discreetly in a secure drop-box located outside or near to the counsellor’s usual room.

The counsellors and researchers delivering the classroom sensitization sessions will be provided with a structured manual and complete a one-day, office-based training. Training will be conducted by master’s level psychologists (who will also serve as supervisors) and comprise lectures, demonstrations, and role-plays. The training will be followed by a period of supervised field practice, when the counsellors and researchers will be required to complete at least two classroom sessions independently under direct observation from supervisors. Fidelity of intervention delivery will be assessed on a checklist of observable procedures which have been distilled from the intervention manual. Each procedure will be rated on a three-point Likert scale (not completed, partially completed, fully completed). A ‘refresher’ training session will also be conducted before the trial begins.

##### Control condition

This will comprise whole-school sensitization activities. The supervisor will meet the principal of each school individually to inform them about planned counselling and research activities and to seek their cooperation for the same. This meeting will also provide structured information about common mental health problems faced by adolescents and address any concerns related to planned procedures and resource demands. Teachers will be invited to participate in separate group sensitization meetings (up to 30 teachers at a time). A standardized script will mirror the topics covered in the meetings with the school principals, but with additional emphasis placed on referral procedures for the host trial. Up to three meetings will be held in each school to maximize coverage of teaching staff. These meetings will be conducted by the same counsellor and researcher pairings responsible for delivering the classroom intervention. Posters will be placed in highly visible locations such as noticeboards or common corridors, in addition to signage on the drop-box, which will remind students (and teachers) of the counselling service.

#### Host trial

##### Intervention arm

A problem-solving intervention will be delivered to individual students across four to five face-to-face sessions spread over 3 weeks. Each session will last for up to 30 min (aligned with the usual duration of school periods) and will be delivered in the local language (Hindi). The sessions will be conducted on school premises in private rooms or, where private rooms are not available, behind screens and curtains in a suitable location (e.g., the school library). Such contingencies to address space limitations were piloted in earlier work and deemed to be feasible and acceptable in the local context, enabling a temporary counselling space in which students would not be on direct view.

Session 1 will focus on fostering engagement, understanding the participant’s difficulties, and introducing the structure and process of the intervention. Over the next three sessions, the participant will be helped to learn and apply a structured problem-solving strategy involving three steps (following the acronym “POD”): (1) identify and prioritize distressing/impairing problems (“*P*roblem identification”); (2) generate and select coping options for modifying the identified problem directly (problem-focused strategies) and/or the associated stress response (emotion-focused strategies) (“*O*ption generation”); and (3) implement and evaluate the outcome of this strategy (“*D*o it”). The intervention may be concluded after four sessions or else extended to a fifth session, depending on the adolescent’s preferences and logistical barriers to intervention completion such as exam breaks and holidays. The concluding session will focus on consolidating learning and generalizing problem-solving skills across different contexts. With permission, all sessions will be audio-recorded for office-based quality and fidelity assessments. Adolescents will be encouraged to practice problem-solving skills between the sessions, aided by a set of three “POD booklets” which explain problem solving using illustrated vignettes and suggest corresponding between-session practice exercises. The booklets (each corresponding to one of the steps of problem solving) will be distributed sequentially over the first three intervention sessions. In the concluding session, the adolescent will be additionally handed a full-color POD poster that summarizes the three steps of problem-solving.

Each school will have one or two counsellors, depending on demand. The counsellors will be Hindi-speaking college graduates aged 18 years or above, with no formal training or qualifications related to psychotherapy or mental health. They will be recruited through online job portals commonly used in the NGO/public sector in India. Selection will be based on reasoning capacity (assessed by written test) and interpersonal skills (assessed by structured role-plays and interview). Selected candidates will receive an intervention manual and complete one week of classroom-based training involving a combination of lectures, demonstrations, and role-plays. This will be followed by a minimum 6-week period of field training in which counsellors will carry out casework (with at least four cases) under the supervision of psychologists. Trainees’ performance will be evaluated using structured role-plays at the end of classroom-based training, as well as supervisors’ ratings of audio-recorded intervention sessions.

Counsellors will participate in weekly peer group supervision meetings, based on an approach tested in the PREMIUM trials, where it was found to be an acceptable, effective, and scalable supervision model for lay counsellors in low-resource settings [39]. Each 2-h meeting will be facilitated by one of the counsellors in rotation and overseen by a supervisor. Counsellors will review and discuss one or two audio-recorded sessions in each meeting. Audio-recordings will be rated by all group members using a therapy quality rating scale that incorporates elements from two established scales [40, 41] and assesses skills specific to problem solving as well as non-specific therapeutic skills (e.g., empathic understanding). Recurrent skills deficits noted by supervisors will be addressed through supplementary training workshops held on a monthly basis. The supervision schedule will ensure a representative selection of audio-recorded sessions, with the intention that all counsellors should receive equal opportunities to discuss their cases. In addition, supervisors will undertake weekly telephone calls (lasting 20–30 min) with each counsellor in order to monitor the progress of their caseload, and identify and manage risks. The counsellors will be able to initiate ad hoc calls if urgent consultation is needed on any case.

##### Control arm

There are no mental health services in the participating schools. A standardized control arm was devised accordingly, keeping in mind the requirement to offer a pragmatic, resource-efficient mode of support with minimal risk of contamination between trial arms. In terms of contamination, a recent scoping review of complex intervention trials in mental health [42] found that the principal processes leading to contamination were the same clinicians treating participants across conditions and communication between clinicians/participants. Moreover, the review recommended that methods other than cluster randomization should be considered to minimize contamination, given the lack of evidence for a link between the level of randomization and intervention effect size.

Participants allocated to the control arm will therefore receive the same printed problem-solving materials used in the intervention arm but without any counsellor contact. Immediately following random allocation to this condition, a researcher (rather than a counsellor) will provide a set of POD booklets and explain their purpose and contents using a standardized script. Students will be encouraged to read through the booklets in sequence and complete the specified practice exercises. No further guidance will be provided. In this way, all trial participants will receive the POD booklets, thereby eliminating the likeliest source of contamination. The counselling process itself is less likely to spill-over as this will be delivered in a one-to-one individual format, and our formative and pilot work showed that students emphasized confidentiality (mentioned earlier) such that disclosure of counselling experiences should be minimized.

### Screening and outcome measures

#### Embedded recruitment trial

The primary outcome (referral rate based on the proportion of referred adolescents as a function of the total sampling frame in each condition) will be collated from referral logs maintained by researchers in each school. Referral data will be aggregated over each 4-week calendar period. Students deemed ineligible for participation in the host trial will be allowed to re-refer themselves after a gap of 4 weeks, offering a suitable time period to re-assess mental health status in line with the host trial’s inclusion criterion about symptom chronicity (Table 1). Secondary outcomes pertaining to the eligibility and clinical characteristics of students referred to the host trial will be derived from screening data on the SDQ (see below).

#### Host trial

All screening and outcome assessments will be undertaken using standardized self-report measures that have been translated into Hindi. Clinical eligibility criteria (i.e., severity, chronicity, and impacts of mental health symptoms) will be assessed using the adolescent-reported form of the SDQ (including the Impact Supplement). The same screening data will also serve as the baseline SDQ/Impact Supplement outcomes for eligible participants who are subsequently enrolled in the trial; baseline assessments for other outcome measures will be completed as soon as possible after completing consent procedures (ideally within 2 working days). The adolescent-reported SDQ/Impact Supplement will be repeated at 6 and 12 weeks post-randomization, along with the parent-reported SDQ/Impact Supplement, and adolescent-reported Youth Top Problems (YTP) [43], Perceived Stress Scale-4 (PSS-4) [44] and Short Warwick-Edinburgh Mental Wellbeing Scale (SWEMWBS) [45]. These measures are described in Table 2. The SDQ will also serve as the basis for assessing remission at both end-points, defined as falling below cut-offs for eligibility on both the SDQ Total Difficulties score and Impact score.

**Table 2:** Outcome measures in the host trial

Measures	Description	Respondent
Primary outcomes at 6 weeks post-randomization		
Strengths and Difficulties Questionnaire (SDQ) Total Difficulties score	The SDQ is the most widely used measure for psychopathology in children and adolescents globally and in South Asia. It has been used in a number of other research studies in India, and has been translated into Hindi and several other Indian languages [34, 46–48]. A Total Difficulties scale score is derived by summing items from four problem subscales (emotional, conduct, hyperactivity/inattention, and peer relationship), while a fifth subscale measures prosocial functioning and does not contribute to the overall severity score. Individual problem scale items are scored from 0 to 2 (with higher scores indicating greater problem severity), giving a range of 0–40. The Total Difficulties score at 6 weeks will be our co-primary outcome	Adolescent-reported
Youth Top Problems (YTP)	The YTP is a brief, idiographic measure on which the respondent identifies, prioritizes, and rates three main problems [43]. Each of the three problems is scored from 0 to 10 according to perceived problem severity (with higher scores indicating greater severity). A mean severity score is calculated by summing individual problem scores and then dividing by the number of nominated problems. The YTP was translated into Hindi for our pilot studies, where it was found to be highly sensitive to change over the course of the problem-solving intervention. The mean YTP score at 6 weeks will be our co-primary outcome	Adolescent-reported
Secondary outcomes over a 12-week period post-randomization^a^		
SDQ Total Difficulties score	(See above for description of the measure)	Adolescent-reported
YTP	(See above for description of the measure)	Adolescent-reported
SDQ Impact Supplement	The SDQ Impact Supplement measures distress and functional impairment associated with index mental health difficulties identified on the main SDQ scale [35]. One item on overall distress and four items on domain-specific functional impairment (home life, friendships, classroom learning, leisure activities) are individually scored from 0 to 2 (with higher scores indicating greater impact), generating a total impact score from 0 to 10	Adolescent-reported
SDQ internalizing subscale	The internalizing symptom subscale score is calculated by adding the score of the peer relationship and emotional problem subscales. The score ranges from 0 to 20	Adolescent-reported
SDQ externalizing subscale	The externalizing symptom subscale score is calculated by adding the score of the conduct and hyperactivity/inattention problem subscales. The score ranges from 0 to 20	Adolescent-reported
Perceived Stress Scale-4 (PSS-4)	The PSS-4 measures perceptions of stress, reflecting the degree to which situations are appraised as stressful during the preceding month. This brief measure was chosen because of its feasibility and relevance as a presumed mechanism of change within the problem-solving intervention, consistent with stress-coping theory. It has been translated into Hindi and used in a number of surveys and as an outcome measure in trials around the world. This brief instrument uses a five-point scale (0 = never, 1 = almost never, 2 sometimes, 3 = fairly often, 4 = very often) to assess how often the respondent has experienced primary appraisals of events as stressful. The total score ranges between 0 and 16, with higher scores indicating a stronger tendency towards stressful appraisals. A study of secondary students in Hyderabad, India reported high internal consistency (Cronbach’s alpha = 0.84) and test-retest reliability (0.85) for the longer 14-item form of the PSS [49]	Adolescent-reported
Short Warwick-Edinburgh Mental Wellbeing Scale (SWEMWBS)	The SWEMWBS is a commonly used measure for mental wellbeing. Wellbeing has been closely linked with social factors such as peer bullying and perception of school connectedness [50]; it may therefore be especially amenable to problem solving. The SWEMWBS is a unidimensional scale that comprises seven items scored on a five-point scale (1 = none of the time, 2 = rarely, 3 = some of the time, 4 = often and 5 = all of the time) with a total range from 7 to 35, where higher scores indicate more positive mental wellbeing. Strong internal consistency has been previously reported in adolescent samples [45]. The measure has been used internationally and a Hindi version is available [51]	Adolescent-reported
Remission^a^	Remission will be defined using the ‘crossing clinical threshold’ method [52] for two clinical criteria (both of which must be met): (i) SDQ Total Difficulties score < 19 for boys or < 20 for girls, and (ii) Impact score < 2	Adolescent-reported
Exploratory outcomes over a 12-week period post-randomization^a^		
SDQ Total Difficulties score	(See above for description of the measure)	Caregiver-reported
SDQ Impact Supplement	(See above for description of the measure)	Caregiver-reported
SDQ internalizing subscale	(See above for description of the measure)	Caregiver-reported score
SDQ externalizing subscale	(See above for description of the measure)	Caregiver-reported score
SDQ prosocial subscale	(See above for description of the measure)	Adolescent-reported score

^a^Repeated measures analysis of 6-week and 12-week endpoints, adjusting for baseline values

### Process measures

Process data on enrollment, randomization, and assessment procedures in both trials will be obtained from researcher-completed record forms. These will be collated to obtain assent/consent rates of adolescents and parents (and reasons for missing assent/consent); randomization rates (and reasons for randomization errors); completion rates of baseline and follow-up outcome assessments (and reasons for non-completion); and time lags between intended and completed assessments (and reasons for deviating from targets). In addition, motivations for help-seeking and expectancies for the school counselling program will be explored at the time of eligibility assessment through a brief qualitative interview with a sub-sample of referred students. Assent/consent to use the interview data in the research will be obtained as part of the consent process for the embedded recruitment trial.

Intervention processes will be assessed using additional data sources. In the embedded recruitment trial, counsellor-completed record forms will provide data on key participation indicators, including attendance rates and duration for all teacher meetings and classroom sensitization sessions, in addition to the numbers of posters and drop-boxes installed in the schools.

In the intervention arm of the host trial, counsellor-completed session record forms will be used to obtain process data on duration, spacing, and frequency of attended sessions (and reasons for non-attendance); and intervention uptake and completion rates (and reasons for pre-intervention and mid-intervention drop-out). Participants’ adherence to intervention activities and potential engagement challenges will be assessed using checklists within the same record forms, indicating whether or not the student completed practice exercises at home; used the POD booklets at home; brought the POD booklets to the session; and demonstrated understanding of POD booklets and session content. Use of POD booklets will be assessed in each arm of the trial at 6- and 12-week follow-up using a brief adolescent-reported measure that asks about estimated frequency of home use and perceived helpfulness of POD booklets in the preceding 6 weeks. Service satisfaction data will also be obtained from participants in each trial arm at 12 weeks using an eight-item service satisfaction questionnaire [53]. Three supplementary questions will elicit open-ended written feedback on the most preferred aspects of the service, potential areas for improvement and recommended changes.

Intervention quality and fidelity will be assessed in both trials using independent ratings of audio-recorded sessions. For the classroom sensitization intervention, 20% of all recordings will be selected at random and rated by a psychologist who is not directly involved with supervision of the intervention providers. A similar approach will be taken with the problem-solving intervention, for which 10% of all audio-recorded sessions will be rated independently. Reliability of the independent raters will be established initially by comparison with intervention quality and fidelity ratings from supervisors (see above).

### Blinding

#### Embedded recruitment trial

The researchers who co-facilitate the classroom sensitization sessions will also record referrals and conduct the host trial eligibility assessments. Blinding of the outcome assessors will therefore not be possible.

#### Host trial

Baseline and outcome assessments will be conducted by separate teams of researchers. All trial investigators, apart from the data manager (BB), will be blind to allocation status until the trial arms are revealed in the presence of both the Trial Steering Committee (TSC) and Data Safety and Monitoring Committee (DSMC). However, unblinding of individual participants may be undertaken if requested by the DSMC (e.g., in case of a serious adverse event).

### Data collection, management, and analysis

#### Data collection

**Fig. 4:**
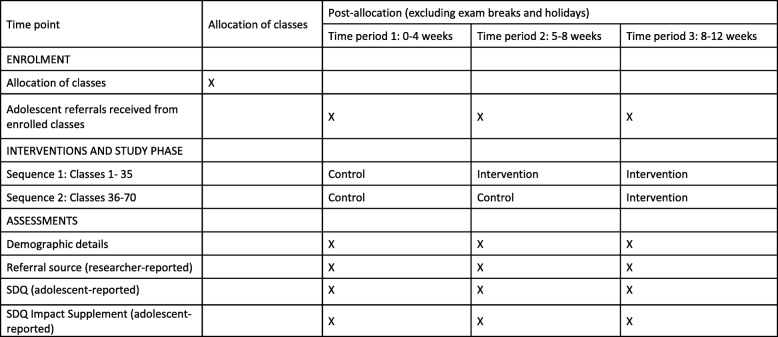
SPIRIT figure for the embedded recruitment trial

**Fig. 5:**
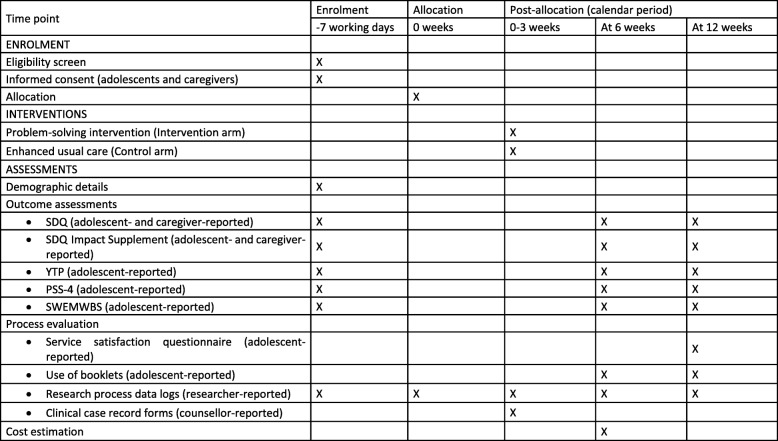
SPIRIT figure for the host trial

There will be a seamless flow of adolescents from the embedded recruitment trial to the host trial. The schedules for enrollment, interventions, and assessments are summarized in separate SPIRIT diagrams for the embedded recruitment trial (Fig. 4) and host trial (Fig. 5). A team of school-based researchers will process the referrals, undertake eligibility assessments for the host trial (within a target of ≤ 3 working days from the date of referral) and obtain adolescent assent/consent (within the same day if possible). A separate team of community-based researchers will visit parents/guardians (within a target of ≤ 2 working days after confirming an adolescent’s eligibility) to obtain consent and complete baseline outcome assessments (within the same day if possible). The school-based research team will complete baseline outcome assessments with adolescents once all consent procedures are completed (within a target of ≤ 2 working days). All assessment procedures should therefore be completed within 7 working days from the date of referral.

The community-based research team (blinded to allocation) will complete follow-up assessments at 6 and 12 weeks post-randomization. Assessments will take place at participants’ homes or other convenient locations, within a maximum period of 7 calendar days from the due date. Researchers will make up to four approaches for each scheduled contact.

Process data from researchers’ logs and counsellors’ session records will be captured on paper forms. All other measures, except for the YTP (which rates idiographic problems and does not readily lend itself to a digital format), will be administered via a tablet computer.

#### Data management

Data will be collected digitally using the customized STAR software program [54], and will be remotely uploaded as comma-separated values (CSV) files on a secured server. The date and time stamps for original data entry will be included, and an audit trail documenting any subsequent changes will be maintained. All paper-based data will be entered manually in SQL Epi-info forms and linked by participant ID with digitally collected data. Range and consistency checks will be performed at weekly intervals, with all inconsistencies and corrections logged to maintain an audit trail. All data will be anonymized and backed-up on external hard disks on a daily basis. All session audio-recordings will be linked with the participant ID and stored in a separate, secure, password-protected folder. A separate password-protected file linking names and participant IDs and the random allocation code will be maintained securely by the data manager and will not be accessed until the unblinding of the trial. All data will be shared in an encrypted form in password-protected files and through secure electronic transfer, when necessary.

#### Data analysis

Quantitative analysis will be conducted using STATA (version 15). A detailed analysis plan will be agreed with the DSMC before any analysis is undertaken. Findings will be reported as per CONSORT guidelines [37] for the host trial, and the CONSORT extension for reporting of stepped-wedge, cluster-randomized trials for the embedded recruitment trial [36].

##### Embedded recruitment trial

The baseline characteristics of the participating 70 classes, including class size and gender composition, will be described and assessed for any systematic differences across the two sequences. The primary outcome will be analyzed using generalized estimating equations (GEE) with robust standard errors. GEE is a recommended method for analysis of stepped-wedge, cluster-randomized controlled trials, providing population-averaged effects of exposure across trial conditions [55]. GEE allows for longitudinal data analysis without resorting to fully specified random effect models and can be applied to both continuous and categorical outcomes [56]. It provides both parameter estimates and standard errors that are corrected for clustering of data and are consistent despite misspecifications in the correlation structure. For this trial, the clustering of data will be specified at the class level. Analysis of the secondary and exploratory outcomes will also be undertaken using the GEE method. Sensitivity analysis will be conducted using a ‘within-period comparison’ of data [38] from the second period only. No interim analyses will be undertaken.

##### Host trial

The trial flowchart will include the number of students referred, screened, eligible, randomized, followed up at 6-week and 12-week endpoints and analyzed for the primary outcomes. The number refusing participation or excluded (with reasons), actively withdrawing, and passively lost to follow-up will be shown by arm. These will be summarized by means (standard deviation), medians (interquartile range), or numbers and proportions as appropriate to relevant subgroups (defined by age, gender, and baseline outcome score). For continuous outcomes, histograms within each arm will be plotted to assess normality and determine whether transformation is required.

The primary analyses will be on an intention-to-treat basis at the 6-week end-point, adjusted for baseline values of the outcome measure; school (as a fixed effect in the analysis) to allow for within-school clustering; counsellor variation (as a random effect); variables for which randomization did not achieve reasonable balance between the arms at baseline; and variables associated with missing outcome data [57]. Analyses of outcomes will be conducted using linear mixed-effects regression models for continuous outcomes with normally distributed errors (e.g., SDQ Total Difficulties score) and generalized (logistic) mixed-effects regression models for binary outcomes (e.g., remission rate). Intervention effects will be presented as adjusted mean differences and effect sizes (ES), defined as standardized mean differences. We will use 95% confidence intervals (CIs) for continuous outcomes, and adjusted odds ratios with 95% CIs for binary outcomes. Additionally, intervention effects for students who receive fewer sessions than prescribed will be estimated using the Complier Average Causal Effect structural equation model [58]. Repeated-measures analysis will be used to analyze data from the two end-points (6 and 12 weeks). Initial models will include an interaction effect between arm and time to allow for differential effects at these two end-points. This will be retained if there is evidence of effect modification by time. No interim analyses of outcomes will be undertaken.

We will explore potential moderators of intervention effects, with respect to a priori defined modifiers (chronicity of mental health difficulties, severity of mental health difficulties, YTP type, and SDQ caseness profile). We will fit relevant interaction terms and test for heterogeneity of intervention effects in regression models. A mediation analysis will be conducted to examine whether the theoretically driven a priori factor (perceived stress at 6 weeks) mediates the effects of the intervention on mental health symptoms and idiographic problems at 12 weeks.

##### Process evaluation

We will undertake descriptive statistical analysis of quantitative process data to explore the differential implementation of intervention procedures. In addition, thematic analysis will be used to code and organize qualitative interview data on service expectancies (assessed prior to enrolment in the host trial) and qualitative written feedback on service satisfaction (assessed at 12-week follow-up in the host trial). Findings from the various data sources will be triangulated and used to develop explanatory hypotheses about potential differences in intervention delivery and participation across schools, subgroups of participants, and providers. Process evaluation findings will be used to facilitate interpretation of the main trial results. The trial statisticians may conduct further analyses to test hypotheses generated from integration of the process evaluation and trial outcome data; these will necessarily be post hoc and identified as such in any subsequent publications.

##### Cost-effectiveness analysis

An economic evaluation will be conducted to estimate the costs and incremental cost-effectiveness of the problem-solving intervention. A combination of top-down and ingredients-based costing approaches will be used to generate cost estimates for the whole package, and for each package component (e.g., counselling sessions and POD booklets), in the intervention and control arms. All costing will be estimated from the providers’ perspective (the schools and the implementing partner Sangath); financial and economic costs will be calculated for all inputs (e.g., materials, training, supervision, staff time, overheads). The cost analysis will assess the costs of setting up and running the interventions; the distribution of costs across different forms of inputs; the unit cost per student/adolescent reached; the cost per additional case remitted; the cost of delivering all activities in intervention schools; and the cost per unit of measure for selected primary and secondary outcomes. We will estimate the incremental cost-effectiveness of the intervention relative to the control condition (enhanced usual care). The cost-effectiveness measure proposed here will be compared to similar school programs in the region and it will inform program replication, scalability, and financial sustainability.

Results will be plotted on a cost-effectiveness plane and presented as cost-effectiveness acceptability curves to show the probability of the intervention being cost-effective at a range of willingness-to-pay threshold levels. A sensitivity analysis will be conducted to take account of uncertainty and imprecision in the measurements.

### Trial governance

Monitoring and governance for both trials will be provided by a Trial Management Group (TMG; comprising senior investigators and project staff involved in day-to-day coordination of research activities), TSC (comprising senior investigators and independent subject experts), and DSMC (a fully independent group with relevant clinical and trials expertise). The TMG and TSC will review trial process indicators (e.g., rates of screening, eligibility, consent, outcome assessments, adverse events) fortnightly and quarterly, respectively. The independent DSMC will meet at the outset of the two linked trials and again at the time of unblinding the trial results, as well as receiving reports of emergent serious adverse events (as per criteria below). Any trial protocol amendments will be agreed and formulated in conjunction with the TSC and DSMC and submitted to relevant Institutional Review Boards for approval.

## Ethics

### Research ethics

Approvals have been obtained from the Institutional Review Boards of Sangath, Harvard Medical School, the London School of Hygiene and Tropical Medicine, and Indian Council of Medical Research. Harvard Medical School is the trial sponsor while Sangath is the implementing agency in India.

### Consent

A two-stage consent process will be used across both trials. To begin, a school-based researcher will provide each referred student with structured verbal and written information about the use of their screening data for research purposes (as part of the embedded recruitment trial), irrespective of their eligibility to take part in the host trial. Students will be able to opt-out from providing any self-reported data for the embedded recruitment trial. Students meeting eligibility criteria for the host trial will be provided with additional structured verbal information and a printed participant information sheet. Assent will be sought for adolescents aged below 18 years and consent will be sought for adolescents who are 18 years or older. For assenting participants aged under 18 years, consent will also be sought from a parent/guardian for participation of the index adolescent and for their own participation in outcome assessments. Consenting adolescents aged 18 years or older will be able to take part without permission from their parent/guardian. We will seek their permission before approaching a parent/guardian about participating in assessments. When approaching the family member of an index adolescent, telephone contact will be initiated by a community-based researcher in the first instance, after which a meeting will be arranged at their home or another convenient location, if agreed.

### Serious adverse events

Serious adverse events (SAEs) include death, life-threatening events, clinical deterioration requiring hospitalization or other specialist treatment, victimization, sexual abuse, and chronic absenteeism and/or drop-out from school. Immediate safeguarding actions will prioritize the safety of participants. This may involve suicide risk assessment, informing stakeholders, facilitating treatment with specialists, and statutory reporting in line with relevant legislation, such as the Protection of Children from Sexual Offences Act 2012 and the Juvenile Justice (Care and Protection) Act 2000 (last amended in 2015).

Each potential SAE will also be assessed for causality by two clinically qualified co-investigators and classified as unrelated, unlikely, possible, probable, or definitely related to trial participation. In the event that consensus is not reached, a third clinical psychologist (independent of the trial) will review the SAE report. Where causality is deemed to be anything other than unrelated to trial participation, the DSMC will advise on further actions such as withdrawal of individual participants, modifications to the trial protocol, continuing without modifications, or suspending/terminating the trial.

## Discussion

This paper describes an integrated protocol that will evaluate the demand for a school counselling program delivering a low-intensity psychological intervention, and the effectiveness of that intervention for school-going adolescents with elevated mental health presentations in New Delhi, India. The interventions used in the host trial and embedded recruitment trial will be provided by lay counsellors, working under the supervision of psychologists, in Government-run secondary schools catering to adolescents from lower socio-economic groups of the city. Concurrent process evaluation and cost-effectiveness analysis will complement the effectiveness findings, generating important evidence relevant to the scaling up of the interventions. To the best of our knowledge, these two trials have no comparable precedent from any low-resource context, and our findings have the potential to inform the design of school-based interventions to address adolescent mental health problems on a large scale in India and other global settings.

An individually randomized design was chosen for the host trial due to the relatively small number of available schools, which ruled out an alternative cluster-randomized design. The inherent risk of contamination associated with individual randomization was minimized by the inclusion of an enhanced usual care control arm, in which participants received the same printed materials as provided in the intervention arm. The augmentation of face-to-face counselling in the intervention arm was not expected to pose a significant risk of spill-over due to the reluctance of participants to share confidential counselling experiences with peers. Moreover, enhanced usual care was designed in such a way that the same delivery agents would not be involved in treating participants across conditions, ruling out another commonly cited source of contamination [42].

The use of a stepped-wedge cluster randomized design for the embedded recruitment trial was also influenced by pragmatic considerations. Formative and pilot work showed that classroom-based sensitization activities had the potential to increase the volume of referrals for school-based counselling. A stepped-wedge design—in which classes formed natural clusters in each school—offered the potential to stagger the roll-out of classroom sessions so that school-based counsellors could accommodate the anticipated flow of referrals within their limited caseload capacity.

Despite the use of contextually adapted sensitization activities, some potentially eligible students (and/or their caregivers) may be unwilling to participate. Reasons for non-participation will be systematically recorded and examined in the embedded process evaluation. We will also seek to address the concerns of referred adolescents who are not eligible for inclusion in the host trial despite a felt need for counselling. In anticipation, we have designed hand-outs with advice on self-management of common problems (such as academic stress). These hand-outs will be distributed to relevant students by the researchers conducting the baseline screening assessments. Another recruitment challenge relates to the academic calendar in the participating schools, which includes frequent breaks for exams, festivals, and other holidays. These scheduling disruptions may require temporary halting of recruitment (for example, so that students are not recruited immediately prior to a long break, as they would not be able to receive the intervention without a delay).

In addition to the publication of our findings in separate papers for each trial, we will share trial outcomes and implications with the participants and other stakeholder groups, including school principals and the local Department of Education in New Delhi. If effective, we will use the process and economic data to model the costs for scaling up the interventions across the school system in New Delhi. This may involve the deployment of counsellors by the state government under the Educational and Vocational Guidance Scheme (EVGC), due for implementation in some sectors of New Delhi starting from the 2018–2019 academic year.

## Trial status

Both trials are registered with ClinicalTrials.gov (host trial, NCT03630471, https://clinicaltrials.gov/ct2/show/NCT03630471; embedded recruitment trial, NCT03633916, https://clinicaltrials.gov/ct2/show/NCT03633916). Recruitment for both trials was initiated on 20 August 2018. We expect to conclude participant recruitment by February 2019 and complete follow-up assessments by June 2019.

## Additional files

Additional file 1: SPIRIT checklist for embedded recruitment trial. (DOC 122 kb)

Additional file 2: SPIRIT checklist for host trial. (DOC 122 kb)

## Data Availability

Data from pilot studies used for arriving at sample size and power calculations can be made available by the corresponding author upon reasonable request.

## Abbreviations

CI: Confidence interval
DSMC: Data Safety and Monitoring Committee
ES: Effect size
LMICs: Low- and middle-income countries
PSS-4: Perceived Stress Scale-4
SAE: Serious adverse event
SD: Standard deviation
SDQ: Strengths and Difficulties Questionnaire
SWEMWBS: Short Warwick-Edinburgh Mental Wellbeing Scale
TSC: Trial Steering Committee
YTP: Youth Top Problems

## References

[1] Belfer ML (2008). Child and adolescent mental disorders: the magnitude of the problem across the globe. J Child Psychol Psychiatry Allied Discip.

[2] Kessler RC, Amminger GP, Aguilar-Gaxiola S, Alonso J, Lee S, Ustun TB (2007). Age of onset of mental disorders: A review of recent literature. Curr Opin Psychiatry.

[3] Holmes EA, Ghaderi A, Harmer CJ, Ramchandani PG, Cuijpers P, Morrison AP (2018). The Lancet Psychiatry Commission on psychological treatments research in tomorrow's science. Lancet Psychiatry.

[4] Morris J, Belfer M, Daniels A, Flisher A, Ville L, Lora A (2011). Treated prevalence of and mental health services received by children and adolescents in 42 low-and-middle-income countries. J Child Psychol Psychiatry Allied Discip.

[5] Collins PY, Patel V, Joestl SS, March D, Insel TR, Daar AS (2011). Grand challenges in global mental health: A consortium of researchers, advocates and clinicians announces here research priorities for improving the lives of people with mental illness around the world, and calls for urgent action and investment. Nature..

[6] Das Jai K., Salam Rehana A., Lassi Zohra S., Khan Marium Naveed, Mahmood Wajeeha, Patel Vikram, Bhutta Zulfiqar A. (2016). Interventions for Adolescent Mental Health: An Overview of Systematic Reviews. Journal of Adolescent Health.

[7] Weisz JR, Krumholz LS, Santucci L, Thomassin K, Ng MY (2015). Shrinking the gap between research and practice: tailoring and testing youth psychotherapies in clinical care contexts. Annu Rev Clin Psychol.

[8] Murray LK, Jordans MJ (2016). Rethinking the service delivery system of psychological interventions in low and middle income countries. BMC Psychiatry.

[9] Bolton P, Lee C, Haroz EE, Murray L, Dorsey S, Robinson C (2014). A transdiagnostic community-based mental health treatment for comorbid disorders: Development and outcomes of a randomized controlled trial among Burmese refugees in Thailand. PLoS Med.

[10] Murray LK, Hall BJ, Dorsey S, Ugueto AM, Puffer ES, Sim A (2018). An evaluation of a common elements treatment approach for youth in Somali refugee camps. Global Mental Health.

[11] Marchette LK, Weisz JR (2017). Practitioner review: Empirical evolution of youth psychotherapy toward transdiagnostic approaches. J Child Psychol Psychiatry.

[12] García-Escalera J, Chorot P, Valiente RM, Reales JM, Sandín B (2016). Efficacy of transdiagnostic cognitive-behavioral therapy for anxiety and depression in adults, children and adolescents: A meta-analysis. Revista de Psicopatología y Psicología Clínica.

[13] Chorpita BF, Daleiden EL, Park AL, Ward AM, Levy MC, Cromley T (2017). Child STEPs in California: A cluster randomized effectiveness trial comparing modular treatment with community implemented treatment for youth with anxiety, depression, conduct problems, or traumatic stress. J Consult Clin Psychol.

[14] Santucci LC, Thomassin K, Petrovic L, Weisz JR (2015). Building evidence-based interventions for the youth, providers, and contexts of real-world mental-health care. Child Dev Perspect.

[15] Ministry of Health and Family Welfare. Implementation Guidelines Rashtriya Kishor Swasthya Karyakram (RKSK) 2018. New Delhi: Ministry of Health and Family Welfare, Government of India; 2018.

[16] Vellakkal S, Patel V (2015). Designing psychological treatments for scalability: The PREMIUM approach. PLoS One.

[17] Nadkarni A, Weobong B, Weiss HA, McCambridge J, Bhat B, Katti B (2017). Counselling for Alcohol Problems (CAP), a lay counsellor-delivered brief psychological treatment for harmful drinking in men, in primary care in India: a randomised controlled trial. Lancet.

[18] Patel V, Weobong B, Weiss HA, Anand A, Bhat B, Katti B (2017). The Healthy Activity Program (HAP), a lay counsellor-delivered brief psychological treatment for severe depression, in primary care in India: a randomised controlled trial. Lancet.

[19] Michelson D, Malik K, Krishna M, Sharma R, Mathur S, Bhat B, et al. Development of a transdiagnostic, low-intensity, psychological intervention for common adolescent mental health problems in Indian secondary schools. Behav Res Ther. 2019; in press. 10.1016/j.brat.2019.103439 PMC7322400 31466693

[20] Parikh R, Michelson D, Sapru M, Sahu R, Singh A, Cuijpers P, et al. Priorities and preferences for school-based mental health services in India: a multi-stakeholder study with adolescents, parents, school staff and mental health providers. Global Mental Health. 2019; in press. 10.1017/gmh.2019.16 PMC6737585 31531228

[21] Parikh R, Sapru M, Krishna M, Cuijpers P, Patel V, Michelson D (2019). "It is like a mind attack": stress and coping among urban school-going adolescents in India. BMC Psychol.

[22] Roy Kallol, Shinde Sachin, Sarkar Bidyut K., Malik Kanika, Parikh Rachana, Patel Vikram (2019). India’s response to adolescent mental health: a policy review and stakeholder analysis. Social Psychiatry and Psychiatric Epidemiology.

[23] Boustani MM, Frazier SL, Becker KD, Bechor M, Dinizulu SM, Hedemann ER (2015). Common elements of adolescent prevention programs: minimizing burden while maximizing reach. Admin Pol Ment Health.

[24] Chorpita BF, Daleiden EL (2009). Mapping evidence-based treatments for children and adolescents: application of the distillation and matching model to 615 treatments from 322 randomized trials. J Consult Clin Psychol.

[25] World Health Organization. Problem management plus (PM+): psychological help for adults in communities exposed to adversity: WHO Kenyan field-trial version 1.0, 2016: World Health Organization; 2016 [Available from: http://www.who.int/iris/handle/10665/205536. Accessed 30 July 2019.

[26] Dias Amit, Azariah Fredric, Cohen Alex, Anderson Stewart, Morse Jennifer, Cuijpers Pim, Sequeira Miriam, Gaude Vithoba, Soares Salvino, Patel Vikram, Reynolds Charles F. (2017). Intervention development for the indicated prevention of depression in later life: The “DIL” protocol in Goa, India. Contemporary Clinical Trials Communications.

[27] Lazarus RS, Folkman S (1984). Stress, appraisal, and coping.

[28] Chiappetta L, Stark S, Mahmoud KF, Bahnsen KR, Mitchell AM (2018). Motivational interviewing to increase outpatient attendance for adolescent psychiatric patients. J Psychosoc Nurs Ment Health Serv.

[29] Notley C, Christopher R, Hodgekins J, Byrne R, French P, Fowler D (2015). Participant views on involvement in a trial of social recovery cognitive-behavioural therapy. Br J Psychiatry.

[30] Michelson D, Day C (2014). Improving attendance at child and adolescent mental health services for families from socially disadvantaged communities: evaluation of a pre-intake engagement intervention in the UK. Admin Pol Ment Health.

[31] Bonevski B, Randell M, Paul C, Chapman K, Twyman L, Bryant J (2014). Reaching the hard-to-reach: a systematic review of strategies for improving health and medical research with socially disadvantaged groups. BMC Med Res Methodol.

[32] Chan A.-W., Tetzlaff J. M., Gotzsche P. C., Altman D. G., Mann H., Berlin J. A., Dickersin K., Hrobjartsson A., Schulz K. F., Parulekar W. R., Krleza-Jeric K., Laupacis A., Moher D. (2013). SPIRIT 2013 explanation and elaboration: guidance for protocols of clinical trials. BMJ.

[33] Madurasinghe VW (2016). Sandra Eldridge on behalf of MRCSG, Gordon Forbes on behalf of the SECG. Guidelines for reporting embedded recruitment trials. Trials..

[34] Bhola Poornima, Sathyanarayanan Vidya, Rekha DorothyP, Daniel Sheila, Thomas Tinku (2016). Assessment of self-reported emotional and behavioral difficulties among pre-university college students in Bangalore, India. Indian Journal of Community Medicine.

[35] Goodman Robert, Ford Tamsin, Simmons Helen, Gatward Rebecca, Meltzer Howart (2000). Using the Strengths and Difficulties Questionnaire (SDQ) to screen for child psychiatric disorders in a community sample. British Journal of Psychiatry.

[36] Hemming K, Taljaard M, McKenzie JE, Hooper R, Copas A, Thompson JA, et al. Reporting of stepped wedge cluster randomised trials: extension of the CONSORT 2010 statement with explanation and elaboration. BMJ. 2018;363:k1614 10.1136/bmj.k1614 PMC6225589 30413417

[37] Moher D, Hopewell S, Schulz KF, Montori V, Gøtzsche PC, Devereaux PJ (2010). CONSORT 2010 Explanation and Elaboration: updated guidelines for reporting parallel group randomised trials. BMJ..

[38] Thompson J.A., Davey C., Fielding K., Hargreaves J.R., Hayes R.J. (2018). Robust analysis of stepped wedge trials using cluster-level summaries within periods. Statistics in Medicine.

[39] Singla Daisy R., Weobong Benedict, Nadkarni Abhijit, Chowdhary Neerja, Shinde Sachin, Anand Arpita, Fairburn Christopher G., Dimijdan Sona, Velleman Richard, Weiss Helen, Patel Vikram (2014). Improving the scalability of psychological treatments in developing countries: An evaluation of peer-led therapy quality assessment in Goa, India. Behaviour Research and Therapy.

[40] Kohrt Brandon A., Jordans Mark J.D., Rai Sauharda, Shrestha Pragya, Luitel Nagendra P., Ramaiya Megan K., Singla Daisy R., Patel Vikram (2015). Therapist competence in global mental health: Development of the ENhancing Assessment of Common Therapeutic factors (ENACT) rating scale. Behaviour Research and Therapy.

[41] Muse Kate, McManus Freda, Rakovshik Sarah, Thwaites Richard (2017). Development and psychometric evaluation of the Assessment of Core CBT Skills (ACCS): An observation-based tool for assessing cognitive behavioral therapy competence. Psychological Assessment.

[42] Magill N, Knight R, McCrone P, Ismail K, Landau S. A scoping review of the problems and solutions associated with contamination in trials of complex interventions in mental health. BMC Med Res Methodol. 2019;19(1):4 10.1186/s12874-018-0646-z PMC6323722 30616508

[43] Weisz John R., Chorpita Bruce F., Frye Alice, Ng Mei Yi, Lau Nancy, Bearman Sarah Kate, Ugueto Ana M., Langer David A., Hoagwood Kimberly E. (2011). Youth top problems: Using idiographic, consumer-guided assessment to identify treatment needs and to track change during psychotherapy. Journal of Consulting and Clinical Psychology.

[44] Cohen Sheldon, Kamarck Tom, Mermelstein Robin (1983). A Global Measure of Perceived Stress. Journal of Health and Social Behavior.

[45] Clarke A, Friede T, Putz R, Ashdown J, Martin S, Blake A, et al. Warwick-Edinburgh Mental Well-being Scale (WEMWBS): Validated for teenage school students in England and Scotland. A mixed methods assessment. BMC Public Health. 2011;11:487. 10.1186/1471-2458-11-487 PMC3141456 21693055

[46] Srikala Bharath, Kumar KishoreK.V (2010). Empowering adolescents with life skills education in schools - School mental health program: Does it work?. Indian Journal of Psychiatry.

[47] Srinath Shoba, Kandasamy Preeti, Golhar Tejas S (2010). Epidemiology of child and adolescent mental health disorders in Asia. Current Opinion in Psychiatry.

[48] Singh Kamlesh, Junnarkar Mohita, Sharma Soumya (2015). Anxiety, stress, depression, and psychosocial functioning of Indian adolescents. Indian Journal of Psychiatry.

[49] Augustine LF, Vazir S, Rao SF, Rao MV, Laxmaiah A, Nair KM. Perceived stress, life events & coping among higher secondary students of Hyderabad, India: a pilot study. Indian J Med Res. 2011;134:61–8. PMC3171919 21808136

[50] Patalay Praveetha, Fitzsimons Emla (2016). Correlates of Mental Illness and Wellbeing in Children: Are They the Same? Results From the UK Millennium Cohort Study. Journal of the American Academy of Child & Adolescent Psychiatry.

[51] Stewart-Brown Sarah (2012). The Warwick-Edinburgh Mental Well-Being Scale (WEMWBS): Performance in Different Cultural and Geographical Groups. Mental Well-Being.

[52] Wolpert, M., Görzig, A., Deighton, J., Fugard, A. J., Newman, R., & Ford, T. 2015. Comparison of indices of clinically meaningful change in child and adolescent mental health services: difference scores, reliable change, crossing clinical thresholds and ‘added value’-an exploration using parent rated scores on the SDQ. Child and Adolescent Mental Health, 20(2), 94–101. 10.1111/camh.12080 32680384

[53] Larsen Daniel L., Attkisson C.Clifford, Hargreaves William A., Nguyen Tuan D. (1979). Assessment of client/patient satisfaction: Development of a general scale. Evaluation and Program Planning.

[54] OPSPL. STAR: Sangath digital tool for advanced research. 2013.

[55] Barker D, McElduff P, D'Este C, Campbell MJ. Stepped wedge cluster randomised trials: a review of the statistical methodology used and available. BMC Med Res Methodol. 2016;16:69. 10.1186/s12874-016-0176-5 PMC4895892 27267471

[56] LIANG KUNG-YEE, ZEGER SCOTT L. (1986). Longitudinal data analysis using generalized linear models. Biometrika.

[57] Sullivan Thomas R, White Ian R, Salter Amy B, Ryan Philip, Lee Katherine J (2016). Should multiple imputation be the method of choice for handling missing data in randomized trials?. Statistical Methods in Medical Research.

[58] Peugh James L., Strotman Daniel, McGrady Meghan, Rausch Joseph, Kashikar-Zuck Susmita (2017). Beyond intent to treat (ITT): A complier average causal effect (CACE) estimation primer. Journal of School Psychology.

